# Low-Light Monocular Depth Estimation Algorithm Based on Illumination Adaptive Image Enhancement

**DOI:** 10.3390/s26103002

**Published:** 2026-05-10

**Authors:** Xiaoqian Cao, Yang Wang, Wanyu Li, Weifeng Liu

**Affiliations:** 1 School of Electronic and Control Engineering, Shaanxi University of Science and Technology, Xi’an 710021, China; 230612042@sust.edu.cn (W.L.); liuwf@sust.edu.cn (W.L.); 2 School of Electromechanical and Automation, Huaqiao University, Xiamen 361021, China; 25011080020@stu.hqu.edu.cn

**Keywords:** depth estimation, low-light scene, illumination inconsistent correction, image enhancement

## Abstract

Depth estimation in low-light scenes is an enormous challenge in the field of monocular depth estimation. Although numerous algorithms have attempted to improve their performance in low-light scenarios through a variety of techniques, the inconsistent illumination issue caused by local intense or colored light is rarely taken into consideration. To tackle this problem, we proposed an illumination adaptive image enhancement-based low-light depth estimation algorithm (IAIE_LDE) in this paper. Our main contribution is an S-shaped illumination estimation basis illumination adaptive consistent correction model, which is designed to eliminate the edge blurring and depth hole effects in depth maps caused by inconsistent lighting. Meanwhile, a low-light depth estimation architecture consisting of three modules, namely, illumination adaptive correction, low-light image enhancement and depth estimation modules, is constructed and trained. Specifically, the first sub-module is designed to alleviate the illumination inconsistency utilizing the proposed S-shaped illumination adaptive correction model by calculating the corresponding correction coefficients for each pixel according to the estimated illumination; the core module of the classic EnlightGAN algorithm is adopted in the second sub-module to improve the overall brightness of the image and solve the other problems caused by low light; the ZoeDepth model is chosen as our depth estimation sub-module to output a depth map comparable to high-quality illuminated images. Extensive experiments on the widely used Oxford RobotCar and nuScenes datasets indicate superior performance of our method by comparing it with state-of-the-art low-light depth estimation algorithms such as RNW, STEPS, ADDS-DepthNet, and ACDepth, both qualitatively and quantitatively.

## 1. Introduction

Accurate perception and reconstruction of the 3D working space is a crucial issue in applications such as autonomous driving, robot navigation and augmented reality. Among many 3D reconstruction methods, including stereo, SFM, Lidar, etc., monocular depth estimation algorithms have received extensive attention, benefiting from their advantageous low cost and ease of use quality, especially when their performance is significantly improved by the adoption of deep neural networks [1,2,3].

Large-scale datasets, like NYU depth [4], KITTI [5], SceneFlow [6], etc., ensure that increasingly complex networks can be adequately trained and achieve superior performance. Those algorithms based on classic CNN architectures and their enhanced models laid the foundation for deep learning monocular depth estimation algorithms and initiated a research boom in monocular depth estimation algorithms [7,8,9,10,11]. The application of the Transformer architecture and its variants in the field of depth estimation have significantly improved the effectiveness of depth estimation, enabling it to be comparable to other time-consuming and expensive algorithms to a certain extent [12,13,14]. The adoption of generative models has optimized the visual effect of estimated depth maps and the generalization ability of those algorithms [15,16]. To sum up, thanks to the continuous emergence of large-scale datasets and persistent optimization of neural network architectures, deep learning-based monocular depth estimation algorithms have been remarkably improved, demonstrating astonishing performance in recent years [17,18,19,20,21,22].

However, all of the above achievements are contingent upon one condition: all the input images used for training and testing/application are high-quality images obtained under good lighting conditions. Once this condition is violated, for instance, in low-light scenarios, the algorithms’ performance drops sharply for the obvious reason that the previously established mapping relationships between pixels’ brightness, color, edges, etc., and their depth values are disrupted. Unfortunately, this issue cannot be avoided, as it will seriously hinder the all-day application of monocular depth estimation algorithms in fields such as autonomous driving and underground robot navigation.

In view of this, low-light depth estimation (LDE) algorithms have become a new research hot spot in this field, and numerous representative algorithms have emerged simultaneously. Domain adaptation-based algorithms diminish the distribution differences between low-light images and well-lit images and thereby improve the performance of the depth estimation algorithm in low-light scenarios by learning robust illumination representations, reducing image style differences, and aligning distribution features [23,24]. Algorithms based on low-light image enhancement improve the quality of low-light images by means of histogram equalization, adjustment of image dynamic range, and estimation of light components, enabling these images to be comparable to well-lit images and consequently obtaining satisfactory depth estimation results [25,26,27]. In addition, adding auxiliary sensors or lighting facilities that can provide supplementary information for visible cameras, for example, event cameras, single-photon cameras, patterns projected by high definition, etc., is also a commonly adopted option [28,29].

Nevertheless, it is truly a matter of regret that most of the methods focus on the overall performance in low-light scenarios, while non-uniform lighting problems that are common in environments such as night and underground navigation are rarely taken into account [30,31], which, on the contrary, will seriously affect the effect of image enhancement and depth estimation in low-light scenarios. To tackle this problem, especially the more intractable problem in scenes with regionally intense or colored light sources, we propose an illumination adaptive image enhancement-based low-light depth estimation algorithm (IAIE_LDE). Its core idea is to adaptively correct the illumination components of low-light images pixel by pixel for each channel through a meticulously designed S-shaped illumination consistency correction model on the basis of the accurately estimated illumination component. Furthermore, in order to completely solve the LDE problem, an innovative network architecture consisting of three modules—illumination adaptation correction, low-light image enhancement, and depth estimation—is designed. The specific architecture and theoretical basis will be elaborated in detail in the third section. Extensive experiments on the widely used Oxford RobotCar datasets indicate the superior performance of our method by comparing it with state-of-the-art low-light depth estimation algorithms such as STEPS, ADDS-DepthNet, and ACDepth, both qualitatively and quantitatively.

In general, our main contribution in this paper is summarized as:

(1) Propose an illumination estimation-based, S-shaped illumination consistency correction model to eliminate the impact of uneven illumination, especially local intense or colored light sources in low-light scenarios, on depth estimation algorithms.
(2) Establish a monocular depth estimation framework consisting of three modules, including illumination adaptive correction, image enhancement and depth estimation, to solve depth estimation problems in low-light scenes with non-uniform lighting conditions, especially those scenes with local intense or colored light sources.

## 2. Related Work

- **Monocular depth estimation (MDE)**

Deep learning methods and large-scale datasets have laid the foundation for vigorous development of monocular depth estimation algorithms. Eigen et al., as the pioneer, first proposed a multi-scale fusion network to regress the depth [7]. Following on this, subsequent algorithms enhanced the performance of the algorithm by adding priors or optimizing the objective function [9,10]. Wang et al. proposed a monocular self-supervised depth estimation algorithm through optical flow estimation, pose estimation and image swapping based on videos, which is relatively simpler and easier to implement compared to the binocular method [11]. SwinDepth [13] further enhanced monocular depth estimation performance by employing a convolution-free Swin-Transformer as an image feature extractor and proposing a Densely Cascaded Multi-scale Network (DCMNet) that connects every feature map directly to another from different scales via a top-down cascade pathway. LiteMono [14] combines a CNN and Transformer to construct a light-weight self-supervised monocular depth estimation model, which significantly improves efficiency while maintaining the algorithm’s performance at the same time. ZoeDepth [32] was the first approach to combine relative depth estimation and metric depth estimation, leading to a model with excellent generalization performance while maintaining metric scale. CGANs [15] proposed an algorithm that employs an enhanced conditional GAN model with a generator that adopts a network structure similar to UNet and a novel feature up-sampling module to overcome the limitations of insufficient training data diversity and overly blurred depth estimation contours. DepthGAN [16] generated a depth map using a semantic layout as input to aid the construction and manipulation of well-structured 3D scene point clouds. DepthAnything [33] attempted to build a simple yet powerful foundation model that can deal with any images under any circumstances by designing a data engine to automatically collect and annotate large-scale unlabeled data, significantly enlarging the data coverage and reducing generalization error. After the algorithm achieved astonishing results on public datasets, in recent years, the focus of monocular depth estimation research has shifted to optimizing the generalization ability of the algorithm, where depth estimation in low-light scenarios is one of the key aspects [34,35,36,37].

- **Low-light Depth Estimation (LDE)**

The reason why depth estimation algorithms that perform astonishingly well under good lighting conditions suffer a significant performance decline in low-light scenarios is self-evident. On the one hand, from the perspective of deep learning theory, the RGB–depth mapping relationship established for well-lit images is disrupted due to the distribution differences between low-light images and well-lit images; on the other hand, from the perspective of image quality, images captured by visible cameras in low-light scenarios suffer from severe information loss and additional noise because of extremely insufficient exposure. With this as the motivation, researchers have made efforts in various aspects, such as domain adaptation, auxiliary equipment, and low-light image enhancement, in order to improve the quality of the depth estimation algorithm in low-light scenarios. DNA-Depth [38] proposed a day–night adaptation method that simply uses the Fourier transform to address the domain alignment problem between day and night datasets. AGDF-Net [39] designed a novel domain generalization depth feature extraction network with adaptive guidance fusion to fully acquire essential depth features that that are domain generalizable at multi-scale feature levels. SRNSD [40] attempted to improve the domain adaptation ability of depth estimation algorithms in low-light scenarios by incorporating three aspects of constraints, including feature and depth domain adaptation, image perspective constraints, and cropped multi-scale consistency loss. ADDepth [41] leverages CoMoGAN to transform daytime images into nighttime scenes in order to obtain a large-scale paired dataset for low-light scenarios. Although domain adaptation-based methods have improved the performance of depth estimation algorithms in low-light scenarios to a certain extent, the low contrast, additional noise, and uneven illumination problems in low-light images fundamentally hinder further improvements in algorithm performance. ACDepth [42] addressed the low-light depth estimation problem via adaptive contrast learning combined with domain adaptation, presenting a robust monocular depth estimation method from the perspective of high-quality training data generation and domain adaptation. PCDepth [28] utilizes event cameras to provide supplementary information for low-light scenarios and proposes a pattern-based complementary learning architecture to guarantee that attractive complementarity mainly impacts high-level patterns that only occupy a few pixels. LED [43] introduced a cost-effective approach that harnesses a pattern projected by high-definition headlights available in modern vehicles. RNW [25] attempted to address LDE problems with three improvements, including prior-based regularization, mapping-consistent image enhancement and statistics-based mask. STEPS [27] constructed a new architecture that achieves low-light enhancement and depth estimation simultaneously by the use of a newly proposed uncertain pixel masking strategy.

Despite the above, compared with common problems of depth estimation in low-light scenarios, like information loss, additional noise, distributional difference, etc., the issue of uneven illumination that is prevalent in applications, such as autonomous driving at night and robot navigation in underground environments, has received relatively little attention, which is also one of the key factors that affect the quality of depth estimation and subsequent applications. Specifically, brightness saturation caused by local strong light sources and exposure imbalance leads to local weak textures and blurred edges, which subsequently result in phenomena such as black holes, voids, and artifacts in depth maps; the presence of local colored light sources alters the color constancy assumption, disrupts the continuity of the original scene, and leads to incorrect depth edge estimations.

Therefore, we focus our efforts on the problem of depth estimation in non-uniformly illuminated low-light scenarios, particularly those with local intense light sources and local colored light sources. An illumination adaptive image enhancement-based low-light depth estimating algorithm is proposed in this paper to eliminate the influence of local illumination inconsistency on the depth estimation results through a carefully designed illumination estimation basis S-shaped illumination adaptive correction model. The IAIE_LDE network consists of the three modules: illumination adaptive correction, low-light image enhancement and depth estimation.

## 3. Proposed Method

To tackle the depth estimation problem in low-light scenes with uneven illumination conditions, especially those with local intense or colored light sources, we propose an illumination adaptive image enhancement-based low-light depth estimation algorithm (IAIE_LDE) in this paper. This section provides a detailed explanation of the overall architecture and individual components of the proposed algorithm.

### 3.1. Overall Architecture

**Figure 1:**
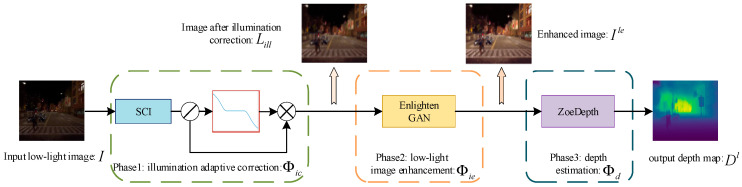
The overall architecture of the proposed algorithm.

As shown in Figure 1, the proposed IAIE_LDE network decomposes the entire depth estimation process into three phases, namely, illumination adaptive correction, low-light image enhancement and depth estimation.

In the first (also most important) stage, image $I\in R^{n\times m\times 3}$ captured in a low-light scene undergoes illumination consistency correction by a novel proposed S-shaped model according to its illumination map $L_{ill}\in R^{n\times m\times 3}$ to address issues related to local lighting inconsistencies. The illumination consistent correction section can be formulated as:

(1) $$I^{ic}=\Phi_{ic}(I|L_{ill})$$

where $I^{ic}\in R^{n\times m\times 3}$ is the image after illumination consistent correction and $\Phi_{ic}$ stands for the entire first module that output the corresponding illumination-corrected image for the original input image. $L_{ill}\in R^{n\times m\times 3}$ is placed here because it is the key reference for illumination correction to calculate the correction coefficient for each pixel in the image. This stage, the most important component in the whole work, ensures that images input to the next phase have a uniform illumination distribution, which is crucial for the subsequent image enhancement and depth estimation stage.

The EnightGAN [44] module is embedded in the following low-light image enhancement stage to improve the illumination-corrected image’s visibility while reducing its noise. This enhancement process amplifies those features that are necessary for depth estimation, such as edges and textures, making the depth estimation results more reliable. The image enhancement section can be formulated as:

(2) $$I^{le}=\Phi_{ie}(I^{ic}|L_{ill})$$

where $I^{le}\in R^{n\times m\times 3}$ is the image after low-light image enhancement and $\Phi_{le}$ stands for the entire low-light image enhancement module. $L_{ill}\in R^{n\times m\times 3}$ is still placed here because it serves as one of the crucial pieces of information required for the image enhancement EnlightGAN [44] model to build the generator.

At last, the Zoe-depth module is adopted and conducted on the previously enhanced image $I^{le}$ to obtain the final depth estimation result. By the end, a performance-efficient depth map $D^{l}\in R^{n\times m}$, which is comparable with that gained in good lighting conditions, can be obtained, even in uneven illumination, low-light scenarios with local intense or colored light sources. The final depth estimation section can be formulated as:

(3) $$D=\Phi_{d}(I^{le})$$

where $\Phi_{d}$ stands for the entire depth estimation module. $D^{l}\in R^{n\times m}$ is the estimated depth map of the input low-light image.

In general, the input–output relationship of the entire network architecture is described as:

(4) $$D=\Phi_{d}(\Phi_{ie}(\Phi_{ic}(I|I_{ill})))$$

where $\Phi_{ic}$, $\Phi_{ie}$, and $\Phi_{d}$ successively represent the three sub-modules of illumination adaptive correction, low-light image enhancement and depth estimation. Formula (4) illustrates the image flow relationship of the entire network: namely, the low-light image is input to the illumination correction sub-module to eliminate the influence of uneven lighting in the first place; after that, the illumination-corrected image is input to the second image enhancement sub-module to improve the overall brightness of the image and solve the other problems caused by low light; and finally, a depth map comparable to high-quality illuminated images can be acquired when the enhanced image is input to the depth estimation sub-module.

### 3.2. Illumination Adaptive Correction Module

The core idea of our proposed IAIE_LDE algorithm, illumination adaptive correction, will be elaborated in this section, including motivation, principle and structure.

The core reason why non-uniform illumination causes difficulties in depth estimation for low-light scenes is that the original rich information of the scene, the assumption of continuous illumination and color constancy are all disrupted due to local overexposure, local coloring, etc. As a result, the depth maps output by existing low-light scene depth estimation algorithms have phenomena such as black holes, holes, and artifacts. Therefore, eliminating this non-uniformity in illumination before image enhancement for low-light image is crucial to solve the problem. Intuitively, simple and easy-to-use algorithms such as histogram equalization are the first choice, but for various low-light conditions with strong or colored light sources in small local areas, simple histogram equalization would significantly change the scene’s illumination distribution and introduce a large amount of noise. Therefore, we aimed to simply correct the consistency of the illumination components without changing the inherent distribution characteristics of the scene. Consequently, separating the illumination components in the input image from the reflection components is the primary task. For this task, various illumination estimation models based on Retinex theory were considered. Among them, the SCI [45] model, which performed exceptionally well in low-light image enhancement and had been well-trained, became our choice.

The illumination consistency correction model is the most important issue and thus should be considered subsequently. Although histogram equalization for the illumination components seems simple and feasible, due to the huge differences in illumination distribution, global equalization would cause extremely unreasonable and significant changes in illumination. Therefore, based on the estimated illumination component for each pixel and each channel, establishing an illumination correction model with the core strategies of high suppression, low enhancement, and medium preservation is a relatively reliable choice. In view of this, an illumination-based S-shaped illumination adaptive correction model is proposed to correct the illumination of the input low-light image pixel by pixel and channel by channel.

Special Note: Since the problem of uneven illumination caused by colored light sources is one of the main issues that we intend to address, the illumination correction operation needs to be performed channel by channel in the RGB. For the sake of simplicity in expression, we use the subscript “ch” to denote the channel-by-channel operation, where $\mathrm{ch}=\left\{R,G,B\right\}$.

Based on the color constancy hypothesis, the Retinex model breaks down an image into the product of the scene’s inherent reflectance and its ambient illumination, expressed as:

(5) I(i,j)=R(i,j)⋅L(i,j)

For each channel in RGB,

(6) $$I_{ch}(i,j)=R_{ch}(i,j)\cdot L_{ch}(i,j)$$

where I(i,j), $I_{ch}(i,j)$ is the image and its corresponding R, G, B channel image captured by the visual camera, (i,j) is its 2D coordinate, R(i,j), $R_{ch}(i,j)$ is the reflectance component determined by the reflective properties of the scene and each channel, and L(i,j), $L_{ch}(i,j)$ is their illumination part affected by light conditions.

Accurately estimating the illumination component is the foundation for achieving adaptive illumination consistent correction. In our proposed method, a pre-trained SCI sub-module, which improves illumination estimation performance through an iterative self-correction approach, is used to estimate the illumination component of the input image.

The illumination estimation module consists of two illumination estimation sub-modules and one self-correction sub-module. Both of the illumination estimation sub-modules adopt the same ResNet architecture, which is the core of the entire module. The self-correction sub-module is the main innovation of SCI, whose core function is to use the illumination component output by the first illumination estimation sub-module as its input and then calculate the inherent reflectance component to further estimate the illumination component through the next illumination estimation sub-module.

For each channel $I_{ch}(i,j)$ of the input low-light image I(i,j), the illumination estimation sub-module outputs its illumination component $L_{ch}(i,j)$ and its corresponding reflectance component $R_{ch}(i,j)$. The proposed novel S-shaped illumination consistency correction model, as shown in Formula (7), is applied to correct the illumination of each pixel

(7) $$L_{ch}^{c}(i,j)=M_{ch}^{c}(i,j)\cdot L_{ch}(i,j)$$

where $L_{ch}^{c}(i,j)$ denotes the illumination component that has been corrected and $M_{ch}^{c}(i,j)$ is the illumination correction coefficient calculated with the S-shaped illumination correction model for each RGB channel, whose mathematical model is presented in Equation (8).

(8) $$M_{ch}^{c}(i,j)=\left\{\begin{array}{c} 2-\frac{1}{1+\alpha^{2}{\left(L_{ch}(i,j)-l_{a}\right)}^{2}},l_{\min}\leq L_{ch}(i,j)\leq l_{a}\\ 1\;,l_{a}<L_{ch}(i,j)\leq l_{b}\\ \frac{1}{1+\beta^{2}{\left(L_{ch}(i,j)-l_{b}\right)}^{2}},l_{b}<L_{ch}(i,j)\leq l_{\max} \end{array}\right.$$

where:

$$\left\{\begin{array}{c} l_{a}=l_{\min}+\omega_{1}\\ l_{b}=l_{\max}-\omega_{2}\\ \omega_{1}=x_{mim}\cdot (l_{median}-l_{\min})\\ \omega_{1}=x_{\max}\cdot (l_{\max}-l_{median}) \end{array}\right.$$

where α and β are respectively the low-light and overexposure adjustment coefficients, which are the hyperparameters that need to be determined during the training process on the network. $l_{\min}$, $l_{\mathrm{median}}$, and $l_{\max}$ are the minimum, median, and maximum illumination values in the input image. $l_{a}$ and $l_{b}$ represent the lower and upper bounds for reliable illumination, which do not need to be corrected. $\omega_{1}$ and $\omega_{2}$ are the key parameters that determine the upper and lower thresholds, which can be automatically adjusted according to the brightness distribution of the image. $x_{\min}$ and $x_{\max}$ are the corresponding threshold adjustment parameters.

At the end, the illumination-corrected image can be obtained by multiplying the inherent reflectance and the corrected illumination component for each channel, which can be described as:

(9) $$I_{ch}(i,j)=R_{ch}(i,j)\cdot L_{ch}^{c}(i,j)=R_{ch}(i,j)\cdot M_{ch}^{c}(i,j)\cdot L_{ch}(i,j)$$

**Figure 2:**
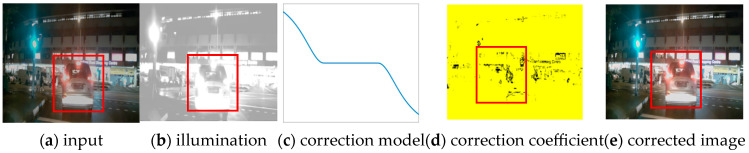
The procedure of illumination adaptive correction.

Figure 2c can greatly assist in understanding the reason for the so-called S-shaped illumination consistency correction model. The design philosophy of the model is easily understood, which is specifically described as follows: For extremely dark pixels, a correction coefficient greater than 1 is assigned; the darker, the bigger. Accordingly, a correction coefficient less than 1 is assigned to super-bright pixels (local light or local color light area); the lighter, the smaller. Of course, the value assigned to middle-light pixels is 1. The criterion to determine whether it is too dark or too light is based on the overall gray-scale distribution of the image.

Figure 2 presents significant images in the illumination adaptive consistent correction phase. Figure 2a is an input low-light image captured on a street at night. Figure 2b is its original illumination map estimated by the SCI sub-module. Figure 2c is the S-shaped illumination correction model illustrating the relationship between the original illumination (abscissa) and the corresponding correction factor (ordinate). Both Figure 2c and Formula (6) indicate that when the original brightness is less than the threshold, the correction coefficient is greater than 1. The lower the brightness, the larger the coefficient, and vice versa. Figure 2d and Figure 2e are respectively the map of the illumination correction coefficient and the image after illumination consistent correction.

### 3.3. Low-Light Image Enhancement

As the second module of our program, the main task of the low-light image enhancement module can be perfectly explained by its name, that is to say: the low-light image enhancement module is designed to improve image visibility and recover structural details that are beneficial to subsequent depth estimation. Theoretically, any low-light image enhancement module with credible performance can be incorporated into our algorithm.

Taking into account the ease of use, performance of the algorithm, and its alignment with the core idea of this paper, the EnlightenGAN [44] module is adopted into our framework because it does not require paired supervised datasets, has excellent edge and texture retention performance, and pays attention to the issue of non-uniform illumination. To be specific, firstly, it does not require paired low-/normal-light supervision, which makes it suitable for real low-light scenes where strictly paired data are difficult to obtain. Secondly, EnlightenGAN can enhance visibility while preserving edge and texture information, thereby providing more reliable geometric cues for the following depth estimation stage. Thirdly, its use of illumination reference (intensity is used in the original model, while it is replaced with illumination component in this paper) and global–local adversarial design is effective for handling spatially inconsistent illumination, which is highly consistent with the uneven lighting problem studied in this paper.

EnlightenGAN is a typical Generative Adversarial Network (GAN); its generator includes an attention-based U-net module, where an attention map, created by normalizing and inverting the original image’s brightness, guides feature extraction. On the other hand, its discriminator consists of a global discriminator for the entire image and a local discriminator for cropped regions, both of which use PatchGAN to reduce local illumination inconsistencies. In the absence of “ground-truth” data, the loss function incorporates global and local discrimination losses, along with structure preservation losses, to maintain overall and local structures between the input and enhanced images.

### 3.4. Low-Light Scene Depth Estimation

Once the low-light image is enhanced, our algorithm utilizes ZoeDepth [18] for depth estimation. ZoeDepth is selected because it combines the advantages of relative depth estimation and metric depth prediction, thus providing strong generalization ability across different scenes. This property is particularly important in our setting since the input image has already undergone illumination correction and enhancement, resulting in a distribution that differs from conventional well-lit inputs. In addition, ZoeDepth can provide stable structural depth recovery without requiring a complete redesign of the depth estimation stage. Because of this, it is adopted as the depth backbone of the proposed framework.

The input image is first processed through the MiDaS encoder to extract features, producing a relative depth map that captures the depth relationships between pixels. Then, the relative depth is converted into absolute depth values, representing actual distances, through the metric head module. These depth values are further calibrated using a 1 × 1 convolution layer and normalized to ensure depth consistency across different scenes. The final output is a depth map containing the actual depth information for each pixel.

### 3.5. Training

Our proposed illumination adaptive image enhancement-based low-light depth estimation network consists of three modules: illumination adaptive correction, low-light image enhancement and depth estimation. The SCI sub-module adopted in the first phase is pre-trained, while the second and third modules retain numerous parameters that need to be optimized through a complex training process.

In order to guarantee performance and training efficiency simultaneously, we divided the entire training process into two stages. At first, the illumination adaptive correction module and the low-light image enhancement module constitute the illumination adaptive low-light image enhancement network, which is trained by a loss function $L_{oss}^{IAIE}$, as written in Formula (10). The IAIE_LDE network, which consists of the well-trained IAIE network in the first training stage and a subsequent depth estimation module, is trained under the constraints of the loss function $L_{oss}^{IALDE}$ in the second stage, which is described in Equation (14).

For the first stage, the enhancement loss is mainly adopted from EnlightenGAN [45]. The loss function $L_{oss}^{IAIE}$, as described in [44] and outlined in Equation (12), primarily consists of four parts: global self-feature preserving loss, local self-feature preserving loss, global generator loss and local generator loss. The global–local joint loss mode is the main contribution that EnlightGAN makes to dealing with regional non-consistent illumination issues in low-light image enhancement.

(10) $$L_{oss}^{IAIE}=L_{SPF}^{l}+L_{SPF}^{g}+L_{G}^{l}+L_{G}^{g}$$

(11) $$L_{SPF}(I^{ic})=\frac{1}{W_{p,q}H_{p,q}}\sum_{i=1}^{W_{p,q}}\sum_{j=1}^{H_{p,q}}{\left(\varphi_{p,q}(I)-\varphi_{p,q}(G(I^{ic}))\right)}^{2}$$

(12) $$L_{G}^{g}=E_{x_{Z}\sim P_{real}}[{\left(D_{Ra}(x_{z},x_{j})-1\right)}^{2}]+E_{x_{j}\sim P_{fake}}[D_{Ra}{\left(x_{j},x_{z}\right)}^{2}]$$

(13) $$L_{G}^{l}=E_{x_{Z}\sim P_{fake\sim patches}}[{\left(D(x_{j})-1\right)}^{2}]$$

where $x_{j}$ and $x_{z}$ represent real and fake samples, respectively, which are drawn from the true and fake distributions. W and H indicate the dimensions of the extracted feature maps, while feature maps extracted from the VGG-16 model are denoted as $\phi_{p,q}()$, where p and q correspond to the q-th convolutional layer following the p-th max pooling layer.

$I^{ic}$ is the low-light image that has undergone illumination consistent correction, and $G(I^{ic})$ refers to the enhanced image output by the generator. $E_{x_{Z}\sim P_{real}}$ represents the average difference values for all real global data, while $E_{x_{Z}\sim P_{fake}}$ represents the average difference values for all fake global data. Similarly, $E_{x_{Z}\sim P_{fake\sim patches}}$ correspond to the average difference values for real and fake local data.

For the second stage, the depth estimation loss mainly follows ZoeDepth and is defined as:

(14) $$L_{oss}^{IALDE}=\frac{1}{A}{\sum_{I^{le}}\left[\log\hat{H}(I^{le})-\log H(I^{le})\right]}^{2}-\frac{1}{2A}{\left(\sum_{f}\left[\log\hat{H}(I^{le})-\log H(I^{le})\right]\right)}^{2}$$

where A is the total number of pixels and $H(I^{le})$ and $\hat{H}(I^{le})$ respectively are the estimated and ground-truth depth values for the enhanced image $I^{le}$. It should be noted that Equation (14) is the main loss function used in the second training stage. The EnlightenGAN-based losses in Equations (10)–(13) are used only for the first-stage enhancement training and are not jointly combined with the ZoeDepth loss in the second stage.

The design of the above loss function aims to balance illumination enhancement, structural preservation, and final depth prediction accuracy. In the first training stage, the enhancement network is supervised by a global–local joint loss. Specifically, the global self-feature preserving loss constrains semantic consistency between the corrected image and the enhanced image at the whole-image level, while the local self-feature preserving loss helps preserve local structures, edges, and textures under spatially inconsistent illumination. In addition, the global and local adversarial generator losses encourage the enhanced image to approach the distribution of visually normal images in both global appearance and local regions. This design is particularly suitable for the low-light scenes considered in this work, where local bright and dark regions often coexist due to illumination inconsistency. In the second training stage, the depth regression loss is introduced to ensure that the corrected and enhanced representations can be effectively transformed into improved depth prediction performance. Therefore, the adopted loss formulation is not chosen merely for complexity but because it matches the characteristics of the proposed multi-stage framework and the target task.

## 4. Experiment

### 4.1. Datasets and Experimental Details

**Datasets**: Our primary training dataset is KITTI. In order to better train the proposed IAIE_LED network, we fine-tuned it using a combination of several other datasets, including HRWSI [46], BlendedMVS [47], ReDWeb [48], DIML-Indoor [49], and MegaDepth [50]. The Oxford RobotCar [51] and Nuscense [52] datasets are used for testing and performance evaluation.

**Evaluation Metrics**: Following the common practice in monocular depth estimation, we adopt several widely used evaluation metrics, including Absolute Relative Error (Abs Rel), Squared Relative Error (Sq Rel), Root Mean Squared Error (RMSE), Logarithmic Root Mean Squared Error (RMSE log), and threshold accuracy metrics: $\alpha_{1}$, $\alpha_{2}$, and $\alpha_{3}$.

Given the predicted depth $d_{i}$ and the ground-truth depth $d_{i}^{*}$ at pixel i, these metrics are defined as follows:

(15) $$\mathrm{Abs}\;\mathrm{Rel}=\frac{1}{N}\sum_{i=1}^{N}\frac{\left|d_{i}-d_{i}^{*}\right|}{d_{i}^{*}}$$

(16) $$\mathrm{Sq}\;\mathrm{Rel}=\frac{1}{N}\sum_{i=1}^{N}\frac{{\left(d_{i}-d_{i}^{*}\right)}^{2}}{d_{i}^{*}}$$

(17) $$RMSE=\sqrt{\frac{1}{N}\sum_{i=1}^{N}{\left(d_{i}-d_{i}^{*}\right)}^{2}}$$

(18) $$RMSE\log=\sqrt{\frac{1}{N}{\sum_{i=1}^{N}\left(\log d_{i}-\log d_{i}^{*}\right)}^{2}}$$

(19) $$\delta_{k}=\%\left(\max(\frac{d_{i}}{d_{i}^{*}},\frac{d_{i}^{*}}{d_{i}})<1.25^{k}\right),\;k=1,2,3$$

where Abs Rel measures the average relative error between the predicted and ground-truth depth values, reflecting the overall proportional deviation of the prediction. Sq Rel further emphasizes larger depth errors and is therefore sensitive to severe local prediction errors. RMSE evaluates the absolute depth error in the original depth space, while RMSE log measures the error in logarithmic space and better reflects relative depth consistency. The threshold accuracy metrics $\alpha_{1}$, $\alpha_{2}$, and $\alpha_{3}$ represent the percentages of pixels whose predicted depth values fall within increasingly relaxed error thresholds, thus reflecting the reliability of the predicted depth under different tolerance levels.

These metrics are adopted because they are widely used in monocular depth estimation and low-light depth estimation studies, which allows fair comparison with existing methods. Moreover, they evaluate the proposed method from complementary perspectives, including relative error, absolute error, scale consistency, and threshold-based prediction accuracy.

**Training Parameters**: The algorithm is implemented using the PyTorch 2.0 framework and runs on an Ubuntu system. Training is performed on an NVIDIA RTX 4090 GPU, with the learning rate set to 1.61 × 10^−4^ and a batch size of eight. Both hyperparameters, α and β, are given a value of 5. With this hardware configuration, the full training process takes approximately 10 h to complete.

**Figure 3:**
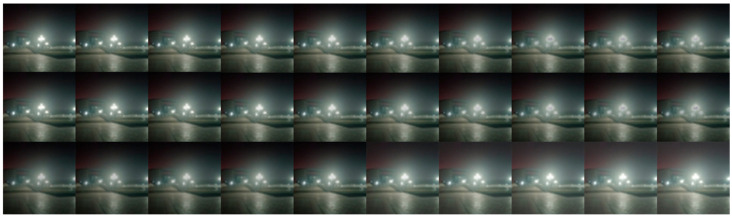
Hyperparameter adjustment comparison.

To validate the effectiveness of the proposed “S-shaped” illumination reliability mask curve and determine the hyperparameters involved, this section experimentally analyzes the influence of the hyperparameters on illumination consistency correction. Based on empirical experience, the value ranges of α and β are set to [1,10]. In theory, if the step size is 1, there are 100 possible combinations of α and β, which would require 100 experiments. To reduce the experimental burden, the following strategy is adopted in this work: first, α and β are adjusted synchronously to determine an optimal common value; then, by fixing α=5 or β=5, the other parameter is varied with a step size of 1 to determine the optimal hyperparameter combination. The final visualization results are shown in Figure 3.

In the first row of Figure 3, α and β are adjusted synchronously from left to right, with both values increasing from 1, 2, …, to 10. It can be observed that when α=β=5, the mask effectively suppresses undesirable illumination while preserving subtle illumination variations, making the transition between bright and dark regions more natural. Therefore, the masking effect is the best under this setting, and α=β=5 is selected.

In the second row of Figure 3, α=5 is fixed, while β increases from 1 to 10. The corresponding illumination consistency correction results show that when β is small, the suppression effect on overexposed regions is almost negligible. However, when β is too large, the suppression of highlight regions becomes overly strong, causing black holes to appear at the centers of light sources.

In the third row of Figure 3, β=5 is fixed, while α=5 increases from 1 to 10. The corresponding correction results indicate that when α is small, the enhancement effect on low-light regions is insufficient, and the overall image remains dark. In contrast, when α is too large, the enhancement in low-light regions becomes excessive, leading to amplified noise and an overall blurry appearance.

Therefore, based on the experimental results and subjective visual assessment, the proposed “S-shaped” illumination reliability curve model achieves the best performance when α=5 and β=5. Accordingly, the optimal hyperparameter setting α=5 and β=5 is adopted in all subsequent experiments.

### 4.2. Ablation Study

**Figure 4:**
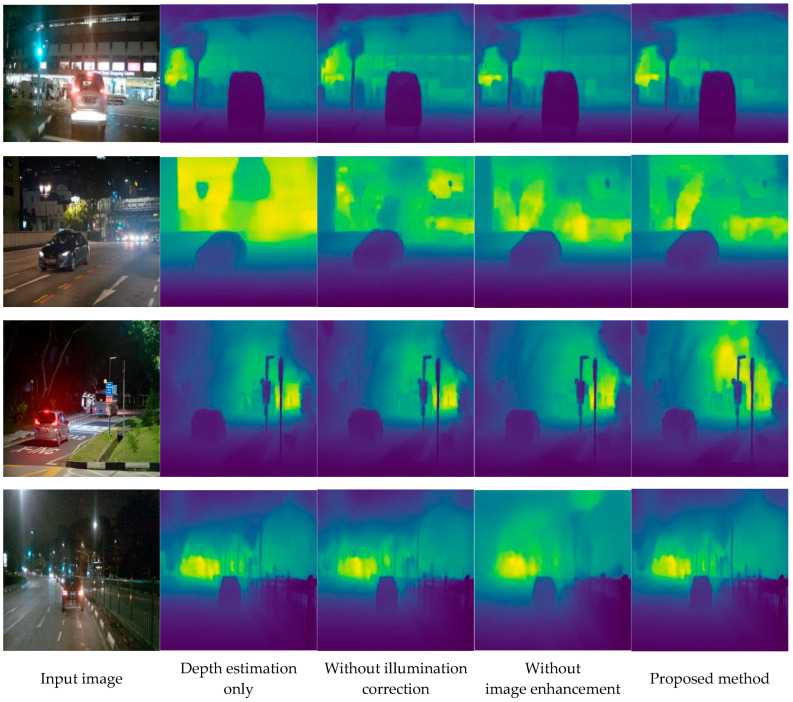
Visual comparison of the ablation study of the proposed IAIE_LDE algorithm.

To evaluate the significance of each component and their contributions, three ablation experiments are conducted: depth estimation only, depth estimation with image enhancement only (no illumination correction), and depth estimation with illumination correction only (no image enhancement). The qualitative and quantitative results are shown in Figure 4 and Table 1 respectively.

In Figure 4, from left to right, the input low-light images and their corresponding depth map estimated by the proposed methods are listed in the first and last column. All the input images in the first column are low-light images with significant uneven illumination caused by a local intense (or colored) light source. The subsequent columns successively exhibit the depth estimation results of the frameworks without correction or an enhancement module and without enhancement and without correction.

Although the visual differences among the ablation variants may appear subtle at first glance, careful examination reveals progressive improvements in specific regions. In the second column (depth estimation only), the depth maps exhibit noticeable edge blurring and structural distortion, particularly around streetlights and road boundaries, confirming that standard depth estimation networks struggle in low-light conditions. The third column (with enhancement but without illumination correction) shows moderate improvement in overall visibility, but the depth maps still contain artifacts in regions with strong local light sources, as the enhancement module alone cannot resolve the illumination inconsistency problem. The fourth column (with illumination correction but without enhancement) demonstrates clearer depth structures, especially in areas affected by non-uniform lighting, highlighting the critical role of the proposed S-shaped correction model. The fifth column (complete method) achieves the best visual quality, with the sharpest object boundaries and most consistent depth gradients across the entire scene.

The visual observations are strongly supported by the quantitative results in Table 1. In Table 1, the symbol ↑ represents the larger the value, the better the algorithm’s performance; conversely, the symbol ↓ represents the smaller, the better. Additionally, the bold data indicate the optimal results in that column. The progressive performance gains across all metrics clearly validate the contribution of each module: Adding image enhancement alone reduces Abs Rel from 0.114 to 0.109, while adding illumination correction alone achieves a more significant reduction to 0.095. The complete framework further reduces Abs Rel to 0.074 and RMSE from 5.908 to 5.082, representing an overall improvement of 35.1% and 14.0%, respectively, over the depth-estimation-only baseline. These consistent improvements across all seven metrics confirm that each module is indispensable, and their combination yields the best performance.

### 4.3. Comparison with State-of-the-Art Approaches

**Figure 5:**
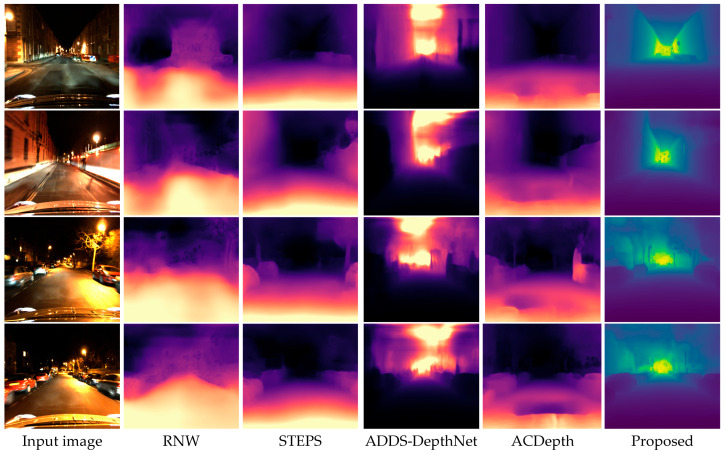
Visual comparison with the state-of-the-art LDE algorithms on the Oxford RobotCar dataset.

**Figure 6:**
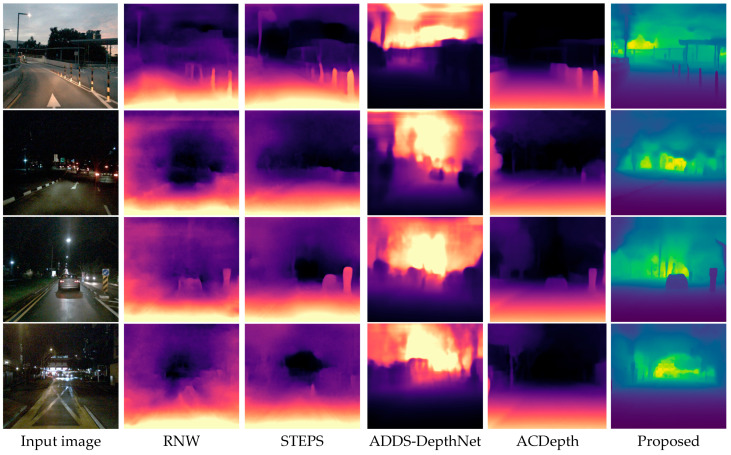
Visual comparison with the state-of-the-art LDE algorithms on the nuScenes dataset.

To evaluate the performance of the proposed method, we compare it with several representative low-light depth estimation approaches, including RNW [25], STEPS [27], ADDS-DepthNet [41], and ACDepth [42]. Qualitative comparisons on different scene images are presented in Figure 5 and Figure 6, while the quantitative results are reported in Table 2. These baseline methods are selected because they correspond to different mainstream technical routes in low-light depth estimation. Specifically, RNW [44] represents nighttime depth estimation with regularization and enhancement-oriented constraints, STEPS [27] performs low-light enhancement and depth estimation in a joint framework, ADDS-DepthNet [41] improves robustness in low-light or all-day scenes through domain adaptation and scene transformation strategies, and ACDepth [42] addresses the low-light depth estimation problem via adaptive contrast learning combined with domain adaptation. Therefore, comparing with these methods covering four distinct technical paradigms allows us to comprehensively assess the effectiveness of the proposed framework from multiple technical perspectives.

In Figure 5, qualitative comparisons on the Oxford RobotCar dataset are presented. From left to right are the input low-light images and depth estimation results of RNW [25], STEPS [27], ADDS-DepthNet [41], ACDepth [42], and our proposed method. The RNW [25] algorithm exhibits substantial depth estimation errors, particularly in darker areas, where object edges appear blurry and the overall depth structure is poorly preserved. The STEPS [27] algorithm performs better than RNW [25], producing relatively smoother depth maps, but still has obvious limitations in edge preservation, especially around vehicle contours and road boundaries. ADDS-DepthNet [41] suffers from severe artifacts in regions with strong local light sources, where the depth maps show significant bright-spot distortions that do not correspond to actual scene geometry, indicating its vulnerability to inconsistent illumination. ACDepth [42] produces depth maps with a more reasonable overall structure than ADDS-DepthNet [41], but it still exhibits noticeable depth discontinuities and blurred boundaries in areas affected by streetlights and vehicle headlights. In contrast, the proposed algorithm (rightmost column) delivers significantly clearer and more accurate depth maps with well-defined road perspective, sharp object boundaries, and consistent depth gradients. The characteristic green-yellow-to-purple color transition in our results accurately reflects the near-to-far depth distribution, demonstrating the effectiveness of the illumination consistency correction module in handling complex nighttime lighting.

In Figure 6, qualitative comparisons on the nuScenes dataset are presented, which includes scenes from different geographic environments with diverse road structures and lighting conditions. The first row shows a dusk scene, while the second and third rows show typical nighttime urban driving scenes. Across all scenes, RNW [25] and STEPS [27] produce depth maps with limited structural detail and blurred edges. ADDS-DepthNet [41] again shows strong sensitivity to local light sources, producing prominent bright artifacts in the depth maps. ACDepth [42] achieves better overall depth estimation than ADDS-DepthNet [41], but the depth boundaries around objects such as vehicles, poles, and road signs remain unclear. The proposed method consistently produces the most visually coherent depth maps, with clear object contours and smooth depth transitions, further confirming its generalization ability across different datasets and driving environments.

Table 2 presents a performance comparison between the proposed algorithm and four baseline methods on both the Oxford RobotCar and nuScenes datasets, where the meanings of symbols “↑, ↓” and bold data are the same as those in Table 1. The data in Table 2 indicate that the proposed algorithm consistently outperforms the baseline algorithms in all metrics, highlighting its effectiveness and superiority for nighttime depth estimation tasks.

On the RobotCar dataset, the proposed method achieves the best performance across all metrics. Compared with the second-best method, STEPS [27], the Abs Rel is reduced from 0.170 to 0.078 (54.1% improvement), and the RMSE is reduced from 6.797 to 2.274. Notably, ACDepth [42] achieves an RMSE of 8.457, even slightly higher than ADDS-DepthNet [41] (8.232).

On the nuScenes dataset, the overall performance of all methods decreases due to the increased scene diversity, but the proposed method still achieves the best results. The a1 score reaches 0.639, outperforming the second-best STEPS [27] (0.528) by 21.0%, and the RMSE is reduced from 10.002 to 6.561. The consistent improvements across both datasets confirm that the proposed method generalizes well beyond a single evaluation scenario.

### 4.4. Runtime Analysis of the Illumination Correction and Enhancement Stage

To evaluate the computational efficiency of the proposed method, this section analyzes the training and inference cost of the network. It should be noted that the main contribution of this work lies in the proposed S-shaped illumination consistency correction model and its integration with the subsequent low-light image enhancement and depth estimation modules to form a monocular depth estimation framework for low-light scenes under non-Lambertian lighting conditions. Among these components, the most direct computational impact is introduced in the front-end illumination correction and low-light enhancement stage. Therefore, the runtime analysis in this section mainly focuses on this stage and compares it with related representative methods.

Table 3 presents the training and testing time of the proposed method in comparison with SCI and EnlightenGAN. As shown in Table 3, after adopting the two-stage training strategy in the enhancement-related stage, the total training time of the proposed method is 2.50 h, which is slightly longer than that of EnlightenGAN (2.23 h) but significantly shorter than that of SCI (4.41 h). In the testing stage, the inference time of the proposed method is 0.62 s, which is comparable to that of EnlightenGAN and clearly lower than that of SCI (1.17 s). These results indicate that the proposed illumination consistency correction mechanism improves performance without introducing a significant additional inference burden.

This result is reasonable. On the one hand, the proposed method preserves the effective illumination estimation mechanism in SCI while designing the illumination consistency correction module as a pixel-wise matrix operation without additional trainable parameters, thereby avoiding the heavy computational burden caused by introducing extra complex network structures. On the other hand, the low-light enhancement stage still adopts EnlightenGAN as the backbone; therefore, the overall testing efficiency remains close to that of EnlightenGAN. In general, the proposed method achieves a favorable trade-off between performance and efficiency in the key innovative stage.

It should also be pointed out that Table 3 reflects the runtime of the front-end stage related to the innovation of this work, rather than the complete end-to-end runtime of the entire low-light depth estimation framework. The overall efficiency of the full system is also affected by the subsequent depth estimation module, and we further discuss this issue in the Discussion Section.

## 5. Conclusions and Discussion

To tackle the low-light depth estimation problem in scenes with uneven illumination caused by local intense or colored light, a novel illumination adaptive image enhancement-based low-light depth estimation algorithm is proposed in this paper. Our main contribution is an S-shaped illumination consistent correction model and an IAIE_LDE network consisting of three modules: illumination correction, image enhancement and depth estimation. The ablation experiments verify the necessity of every part, and comparative evaluations with state-of-the-art algorithms, such as STEPS [27], ADDS-DepthNet [41], and ZoeDepth, using the Oxford RobotCar dataset demonstrate the superior effectiveness of the proposed method in depth estimation tasks.

The proposed method aims to mitigate the adverse effects of illumination inconsistency on monocular depth estimation in low-light scenes, rather than to serve as a dedicated physical denoising model. The illumination consistency correction and enhancement modules can effectively alleviate insufficient visibility in dark regions, local overexposure, and brightness imbalance, providing more reliable inputs for subsequent depth estimation. However, sensor-level noise, such as fixed-pattern noise and dark signal non-uniformity, is not explicitly modeled, and residual noise may be amplified during enhancement in regions with extremely low signal-to-noise ratios.

To evaluate generalizability, experiments are conducted on two datasets with significantly different geographic environments, road structures, and traffic patterns. The proposed method consistently outperforms all baselines on both datasets, and the comparison with representative methods covering different technical paradigms further validates the effectiveness of the proposed framework. Nevertheless, the current evaluation is limited to urban driving scenarios, and robustness under more extreme conditions, such as heavy fog, snow, or indoor environments, requires further investigation.

Regarding computational efficiency, the cascaded multi-stage design inevitably introduces additional overhead compared with single-stage methods. However, the experimental results demonstrate that the illumination correction module achieves meaningful performance gains with minimal additional computational burden, indicating a reasonable trade-off between cost and accuracy.

Despite its effectiveness, the proposed method has several limitations: its performance depends on illumination estimation accuracy, sensor-level degradation factors are not explicitly modeled, the cascaded structure introduces extra computational cost, and the evaluation is limited to urban driving scenarios. In future work, we will explore lighter backbones, shared feature extraction across stages, noise-aware modeling, and extend the evaluation to adverse weather, indoor, and underground scenes.

## Figures and Tables

**Table 1:** Quantitative comparison of the ablation study of the proposed IAIE_LDE algorithm.

	a1↑	a2↑	a3↑	Abs Rel↓	Sq Rel↓	RMSE↓	REMSE_log↓
depth estimation only	0.831	0.915	0.974	0.114	0.147	5.908	0.057
without illumination correction	0.838	0.920	0.979	0.109	0.121	5.731	0.051
without image enhancement	0.847	**0.931**	**0.989**	0.095	**0.097**	5.489	0.054
our proposed algorithm	**0.857**	**0.931**	**0.989**	**0.074**	**0.097**	**5.082**	**0.046**

**Table 2:** Quantitative comparison with the state-of-the-art LDE algorithms.

RobotCar							
	**a1↑**	**a2↑**	**a3↑**	**Abs Rel↓**	**Sq Rel↓**	**RMSE↓**	**REMSE_log↓**
RNW [25]	0.516	0.839	0.923	0.501	3.349	9.957	0.419
STEPS [27]	0.758	0.923	0.968	0.170	1.686	6.797	0.234
ADDS-DepthNet [41]	0.602	0.870	0.902	0.291	3.695	8.232	0.397
ACDepth [42]	0.726	0.898	0.935	0.191	2.488	8.457	0.354
our proposed algorithm	**0.837**	**0.924**	**0.985**	**0.078**	**0.107**	**2.274**	**0.047**
**nuScenes**							
	**a1↑**	**a2↑**	**a3↑**	**Abs Rel↓**	**Sq Rel↓**	**RMSE↓**	**REMSE_log↓**
RNW [25]	0.486	0.727	0.835	0.401	3.757	10.457	0.589
STEPS [27]	0.528	0.755	0.872	0.314	3.583	10.002	0.421
ADDS-DepthNet [41]	0.514	0.803	0.899	0.274	3.259	11.001	0.419
ACDepth [42]	0.519	0.732	0.861	0.326	3.678	10.339	0.461
our proposed algorithm	**0.639**	**0.821**	**0.900**	**0.265**	**2.645**	**6.561**	**0.379**

**Table 3:** Training and inference time comparison for the illumination correction and enhancement stage.

Methods	Training Time/h	Test Time/s
SCI [45]	4.41	1.17
EnlightenGAN [44]	2.23	0.62
Ours	2.50	0.62

## Data Availability

All datasets used in this study are publicly available from their official websites. The implementation details of the proposed method are described in detail in the manuscript, and the related source code will be publicly released on GitHub 1.0 before the article is made public at https://github.com/fififft/zoe (accessed on 1 June 2026.)
